# miRmapper: A Tool for Interpretation of miRNA–mRNA Interaction Networks

**DOI:** 10.3390/genes9090458

**Published:** 2018-09-14

**Authors:** Willian A. da Silveira, Ludivine Renaud, Jonathan Simpson, William B. Glen, Edward. S. Hazard, Dongjun Chung, Gary Hardiman

**Affiliations:** 1 Center for Genomic Medicine, Bioinformatics, Medical University of South Carolina (MUSC), Charleston, SC 29425, USA; silveira@musc.edu (W.A.d.S.); jondsimp@gmail.com (J.S.), glen@musc.edu (W.B.G.); hazards@musc.edu (E.S.H.); 2 Division of Nephrology, Department of Medicine, Medical University of South Carolina (MUSC), Charleston, SC 29425, USA; renaudl@musc.edu; 3 Laboratory for Marine Systems Biology, Hollings Marine Laboratory, Charleston, SC 29412, USA; 4 Academic Affairs Faculty, Medical University of South Carolina (MUSC), Charleston, SC 29425, USA; 5 Department of Public Health Sciences, Medical University of South Carolina (MUSC), Charleston, SC 29425, USA; chungd@musc.edu; 6 Institute for Global Food Security, Queens University Belfast, Stranmillis Road, Belfast BT9 5AG, UK

**Keywords:** bioinformatics pipelines, algorithm development for network integration, miRNA–gene expression networks, multiomics integration, network topology analysis

## Abstract

It is estimated that 30% of all genes in the mammalian cells are regulated by microRNA (miRNAs). The most relevant miRNAs in a cellular context are not necessarily those with the greatest change in expression levels between healthy and diseased tissue. Differentially expressed (DE) miRNAs that modulate a large number of messenger RNA (mRNA) transcripts ultimately have a greater influence in determining phenotypic outcomes and are more important in a global biological context than miRNAs that modulate just a few mRNA transcripts. Here, we describe the development of a tool, “miRmapper”, which identifies the most dominant miRNAs in a miRNA–mRNA network and recognizes similarities between miRNAs based on commonly regulated mRNAs. Using a list of miRNA–target gene interactions and a list of DE transcripts, miRmapper provides several outputs: (1) an adjacency matrix that is used to calculate miRNA similarity utilizing the Jaccard distance; (2) a dendrogram and (3) an identity heatmap displaying miRNA clusters based on their effect on mRNA expression; (4) a miRNA impact table and (5) a barplot that provides a visual illustration of this impact. We tested this tool using nonmetastatic and metastatic bladder cancer cell lines and demonstrated that the most relevant miRNAs in a cellular context are not necessarily those with the greatest fold change. Additionally, by exploiting the Jaccard distance, we unraveled novel cooperative interactions between miRNAs from independent families in regulating common target mRNAs; i.e., five of the top 10 miRNAs act in synergy.

## 1. Introduction

Mature microRNAs (miRNAs) are ~22-nucleotide-long single-stranded noncoding RNAs which function as translational repressors in all known animal and plant genomes [1,2]. It is estimated that 30% of all genes in the mammalian cells are regulated by miRNAs [3]. Each miRNA can regulate the expression of hundreds of messenger RNAs (mRNAs), and each mRNA can be targeted by various miRNAs, with multiple miRNA-binding sites being required for the efficient repression of a target mRNA [2,3].

The traditional paradigm regarding the mode of silencing of miRNAs is that (1) most animal miRNAs bind their target mRNAs with mismatches, promoting repression of mRNA translation with little or no influence on mRNA abundance; and that (2) most plant miRNAs bind their targets with near-perfect complementarity, allowing Ago-catalyzed cleavage and degradation of the mRNA strand [4]. The scientific dogma was that perfect complementarity excluded translational repression because it enabled cleavage, and this contributed to the notion that plant and animal miRNAs behaved in fundamentally different ways. However, several reports have demonstrated that animal miRNAs induce significant degradation of target mRNAs [5,6,7,8] and that translational repression also occurs in plants [9].

These studies initiated a debate regarding the mode of action of miRNAs, a discourse that remains active in the scientific community [10,11,12], highlighting the complexity of miRNA-induced *translational repression and degradation*. This sparked yet more unanswered questions such as “is degradation an independent mechanism by which silencing is accomplished?”, or “is it a consequence of a primary effect on translation?” In 2008, Brodersen et al. suggested that translational repression is the default mechanism by which miRNAs repress gene expression in both animals and plants [9], a study that was followed by a contradicting study by Guo et al. [13] stating that mammalian micRNAs predominantly act to decrease target mRNA levels. This work was based on the fact that only a small fraction of repression observed by ribosome profiling (11–16%) is attributable to reduced translational efficiency, whereas at least 84% of the repression is attributable instead to decreased mRNA levels [13].

Several studies have made important advances in elucidating the relative contributions of translational repression and mRNA degradation by animal microRNAs and have further characterized how translational repression is accomplished: inhibition of translation initiation; inhibition of translation elongation; cotranslational protein degradation; and premature termination of translation [14]. Regarding miRNA-induced mRNA degradation, it appears that the extent of degradation is specified by the mRNA target, and not by the miRNA itself, because the same miRNA can either repress translation or induce mRNA decay in a target-specific manner [6]. It remains unclear why some targets are degraded and others are not. It has been suggested that the number, type, and position of mismatches in the miRNA/mRNA duplex plays an important role in triggering degradation or translational arrest [15]. Although defining how miRNAs mediate their repressive effects has been a controversial subject over the past two decades, current evidence suggests that target mRNA degradation contributes largely to the miRNA-induced silencing effects. Given, however, that many of these studies were conducted *in vitro* with cultured mammalian cells rapidly dividing, it is necessary to confirm this shift in paradigm using other cell types and in *in vivo* studies.

**Figure 1:**
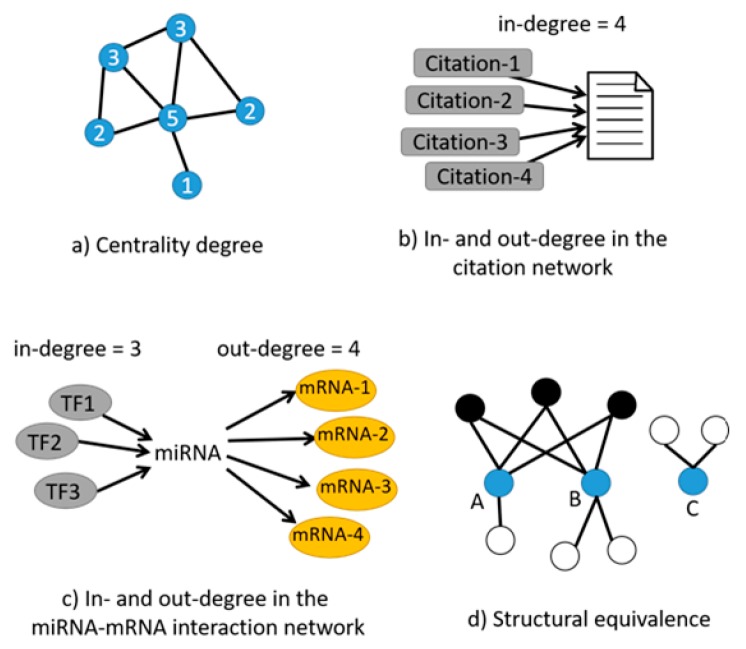
(**a**) The degree of centrality defines the number of edges (black lines) connected to a vertex (blue dots). The number inside the dots represents the centrality degree of each vertex; (**b**) The in-degree of a scientific publication is the number of other papers citing it (citations in grey boxes); (**c**) In a microRNA (miRNA)–messenger RNA (mRNA) interaction network, the number of transcription factors (TF) regulating an miRNA characterizes the in-degree, and the number of mRNA targets of this miRNA for silencing is the out-degree; (**d**) Structural equivalence between 3 vertices, A, B, and C: A and B share, in this case, 3 of the same neighbors (black dots), although both also have other neighbors that are not shared (white dots). Vertex C is not similar to A and B because it does not share any neighbors with them.

In studying networks, including miRNA–mRNA interaction networks, one of the most relevant metrics is “*centrality*”. Simply described, centrality is a measure of the degree, i.e., the number of edges connected to a vertex (Figure 1a) [16]; the assumption is that vertices with the highest degrees (with the most connections) play important roles in the functioning of the system, making the degree of centrality a useful guide for focusing attention on the system’s most crucial elements. In directional networks, vertices have two different degrees, an “in-degree” and an “out-degree”, corresponding to the number of edges pointing inward to and outward from these vertices [16]. In the context of social networks, individuals who have connections to many others may be perceived as having greater influence, more access to information, or higher prestige than those who have fewer connections [17,18]. The same can be applied to the evaluation of scientific publications: the count of how many times a paper has been cited, equivalent to the “in-degree” in the citation network (Figure 1b), provides a measure of whether the paper has been influential or not. This is widely used as a metric for judging the impact of scientific research [19,20]. Centrality, when applied to miRNA–mRNA interaction networks, can highlight which miRNAs are more important than others in a specific context such as disease or biological processes by defining how many in-degrees and out-degrees each miRNA possesses [21,22]; as an example, the number of transcription factors (TF) regulating an miRNA characterizes the “in-degree”, and the number of mRNA targets of this miRNA for silencing is the “out-degree” (Figure 1c), and both metrics can greatly contribute to the determination of the importance of a specific miRNA in a given system [23].

In a network, vertices with an unusually high degree of centrality become “*hubs*”. Even if few hubs exist within a network, they can be very informative and play a central role in the functioning of the system. For example, social networks often contain a few central individuals with many acquaintances. Few websites exist, for instance, with an extraordinarily large number of links. In a cellular context, there are few metabolites that take part in almost all metabolic processes.

Another important metric in network analysis is that of “*structural equivalence*” between vertices, i.e., a measure of *similarity* [16]; two vertices in a network are structurally equivalent if they share many of the same network neighbors (Figure 1d). Online dating sites compute similarity measures to match users to one another by using descriptions of people’s interests, background, likes, and dislikes [24,25]. In the context of miRNA–mRNA interaction networks, measuring structural equivalence could help in identifying groups of collaborative miRNAs based on the number of similar mRNA targets they share [26,27].

### Comparison with Available Tools

According to the increasing experimental evidence supporting target mRNA degradation rather than translational repression as the main silencing mechanism used by miRNAs, the integration of target predictions with miRNA and gene expression profiles based on high-throughput sequencing (HTS) analyses from the same sample would greatly improve the characterization of functional miRNA–mRNA relationships. Several online tools that aim to identify miRNA–mRNA interactions exist: (1) MicroRNA and mRNA integrated analysis (MMIA) [28] is a versatile web server that permits query of miRNA–mRNA interactions. It applies systems level analysis to identify pathways and diseases in which the miRNAs of interest may be involved. However, MMIA ignores the network of collaborative miRNAs that work together to silence genes; (2) **miRror-Suite** [29] uses a list of miRNAs in a contextual manner to predict the most likely set of regulated genes in a cell line or tissue, or from a list of genes. However, the input is either a miRNA list or a gene list, but cannot be both. Additionally, it relies only on public datasets, does not let users provide their own paired miRNA–gene expression datasets, and fails to provide a metric in which miRNA is the most important variable; (3) DIANA-mirExTra [30] uses repository information to build a network with miRNA–gene targets from miRNA and gene expression datasets. However, it does not classify the importance of the miRNA based on interaction (it only considers fold change) and the networks do not provide a metric of miRNA similarity; (4) miRGator [31] is a mining data and hypothesis generating tool that uses big data from public datasets combined with data from miRNA–target repositories and a negative correlation algorithm to define miRNA regulatory networks. It allows enquiries regarding where the expression of the miRNAs is more relevant and the most commonly affected biological functions. However, it does not let users input their own data and lacks biological contextual information for tissue-specific miRNAs; (5) In 2010, the web tool MAGIA (miRNA and genes integrated analysis) was designed, allowing integration of target predictions with gene expression profiles using different relatedness measures for matched and unmatched expression profiles, using miRNA–mRNA bipartite network reconstruction, gene functional enrichment, and pathway annotations for browsing results [32]. In 2012, it was updated to MAGIA^2^, which now focuses on mixed regulatory circuits involving miRNAs, transcription factors (TFs, in-degree measure), and mRNA targets (out-degree measure) [23]. Nevertheless, MAGIA and MAGIA^2^ do not calculate network metrics as degrees of centrality and structural similarity. (6) NetworkAnalyzer [33] is a software plug-in that assesses several network topological parameters, including centrality, and represents them graphically. However, this tool was designed with protein–protein interactions in mind, not miRNA–mRNA networks. Additionally, the shared neighbor measure is not suited to define similarity in miRNA–mRNA networks, and the graphs generated focus on the network parameters and not on the importance, or identification, of each node—i.e., miRNA—in the network. (7) A more recent tool, SpidermiR, allows evaluation of the degree of centrality of miRNA–gene target networks and capabilities that resemble the structural equivalence analysis [34]. Although the graphical output of SpidermiR helps in simplifying network interpretation, the tool is focused on the analysis of public available data, principally from The Cancer Genome Atlas (TCGA) repository, and does not allow the researcher to input locally generated data [34]. None of these tools mentioned above offer a measure for centrality and structural equivalence combined with out-degrees that represent useful metrics to determine the system’s most crucial miRNAs and collaborations between miRNAs. Finally none provide output graphs that help the user focus on these important conclusions (Table 1).

Here, we present miRmapper, an open-source application that researchers can use to identify the most important miRNA and mRNAs, identified in their own experimental design or produced by publicly available data, in a miRNA–mRNA interaction network by leveraging the centrality and similarity metrics. Based on the assumption that miRNAs with the highest number of target genes are probably the most important ones, and that the genes being targeted by numerous miRNAs are probably the most crucial ones, miRmapper users can easily visualize collaborative miRNAs in relation to their mRNA targets as a result of graphical outputs such as dendrograms and heatmaps. This ultimately allows the user to focus their attention on the system’s most crucial elements. Note that miRmapper is designed for miRNA–mRNA interactions in the context of mRNA degradation and measures the centrality, similarity, and out-degree of each miRNA based on the topology of the miRNA–mRNA interaction network created from same-sample miRNA-seq and mRNA-seq datasets. This novel tool will provide information that can drive further research by uncovering potential biomarkers and drug targets.

## 2. Materials and Methods

The method presented here is based on the following assumptions: (1) miRNAs tend to act via the downregulation of their gene targets, in an inverse correlation relationship (i.e., miRNA canonical function) [35]; (2) the regulatory effect of miRNAs is dependent on the cellular context [35,36]; (3) miRNAs regulating the greatest number of targets have a greater impact on the phenotype (network centrality) [16,37]; (4) in a given context, the list of common targets of two miRNAs can be used to infer how similar their effects are, independent of their nucleotide sequence similarity (network similarity by structural equivalence) [16,38]; and (5) a gene being regulated by the greatest number of miRNAs is probably a key gene in the system studied [16,39].

This package was conceived to be used downstream of paired miRNA and differential gene expression analyses, and it also requires a list of interactions of the DE miRNAs and target genes. For sequencing experiments, DE analysis can be performed using DESeq2, EdgeR, and Limma programs for mRNA sequencing [40,41]. DE results for miRNA sequencing can be obtained from the CAP-miRSeq pipeline, mirPRo, and miARma-Seq [42,43,44]. Any form of DE analysis that permits the acquisition of a list of DE mRNAs and DE miRNAs—such as high-throughput sequencing, microarray technology, quantitative PCR (qPCR) arrays, etc.—can be used as input. Predicted targets of miRNA can be collected from databases such as microRNA.org, TargetScan, and the multiMiR R Package [45,46,47]. The user needs to be aware that repositories provide the entire list of predicted genes and that only those that are in the DE gene list are of interest. Packages such as multiMiR have functionalities to select only the appropriate interactions [47]. Consequently, users using an interaction list directly from other repositories will have to use the intersection of genes between the interaction list and the list of DE genes in their experiment. Taking into account our first assumption, we considered the analysis to be more insightful if only downregulated mRNAs are selected as possible targets for upregulated miRNAs and vice versa.

**Figure 2:**
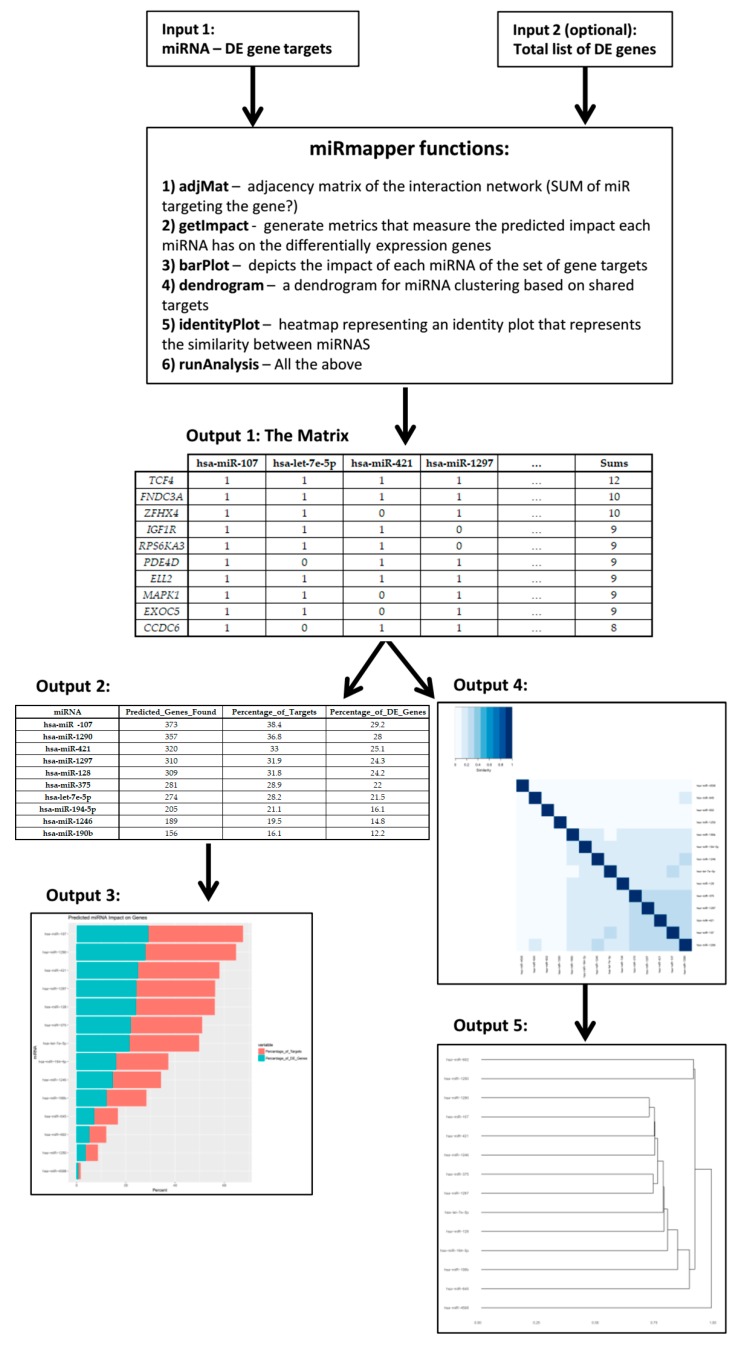
The miRmapper workflow. An miRNA-gene interaction data frame is the required input for the tool (Input 1), additionally a list of total differentially expressed (DE) genes can be used in conjunction (Input 2). The use of the miRmapper functions will provide an adjacency matrix of the miRNA-genes interactions with gene centrality (Output 1), from this a table is generated with the miRNA impact on gene expression (Output 2) and the graphical representation of that impact (Output 3). Also from Output 1, the structural similarity of miRNAs networks is calculated and graphically represented as an identity plot (Output 4) and as a dendogram (Output 5).

miRmapper provides simple and effective metrics to analyze the predicted influence of miRNAs on gene expression; a workflow of the method is shown in Figure 2. Starting with the postulate that DE miRNAs that impact a larger number of DE genes are of greater importance for gene regulation in the context of the experiment [16], the percentage of predicted target genes over the total targets is calculated for each miRNA to indicate its level of centrality (Equation (1)). Similarly, we calculate the proportion of predicted targets for each miRNA relative to all differentially expressed genes (Equation (2)); this second calculation not only provides us with the information about miRNA centrality, but adds the overall impact of the miRNA expression in the regulation of a given gene’s expression. The package also provides as output the degrees of centrality for each gene target.

These calculations are provided in both a tabular form and a bar plot of publication quality. The proportions are given by the following formulas, where *t* is the number of predicted target genes for miRNA *m*, *T* is the number of total gene targets, and *G* is the number of total DE genes:

(1) $$Influence_{DE}^{m}=\frac{t}{T}$$

(2) $$Influence_{Total}^{m}=\frac{t}{G}$$

We represent the predicted interactions in the form of an adjacency matrix. The adjacency matrix is a convenient data structure for detecting miRNAs that target the same genes. We then apply the Jaccard distance formula to measure dissimilarity between miRNAs (Equation (3)) [48,49]. With this metric, we calculate and visualize miRNA clustering with an identity plot and dendrogram for a hierarchical representation, i.e., network similarity. The Jaccard distance is given by the following formula, where $D_{ij}$, also known as the Jaccard distance, is the proportion of gene targets that are not shared between miRNAs *i* and *j* relative to the total number of genes targeted by these two miRNAs:

(3) $$D_{ij}=1-\frac{\left|t_{i}\cap t_{j}\right|}{\left|t_{i}\cup t_{j}\right|},$$

where $t_{i}$ and $t_{j}$ are the genes targeted by miRNAs *i* and *j*, |$t_{i}\cap t_{j}$| is the shared gene targets of $t_{i}$ and $t_{j}$, and $\left|t_{i}\cup t_{j}\right|$ is the total gene targets of $t_{i}$ and $t_{j}$.

The Jaccard index has the advantage in that it only counts the mutual presence of gene targets in its calculations [49]. In the context of multiple DE miRNAs with large and non-overlapping lists of interactions, a method that takes into consideration only the presence of the interactions in a list will be the one with the greater biological meaning. Methods such as the simple matching coefficient and the chi-square statistic will consider two miRNA as being highly similar if they have no common gene target, but have a large list of genes that both do not target [50].

The software is implemented as an R package, “miRmapper”. As input, the package requires a table with miRNAs and their targets and an optional list of the total differentially expressed genes. The tool then produces an adjacency matrix describing all miRNA–target interactions and the additional information of the number of miRNAs regulating each gene, i.e., degree of centrality for the genes. From this matrix is calculated the impact that each miRNA has on the list of genes, i.e., degree of centrality for the miRNAs, and the results are depicted as a boxplot ordered by miRNAs with the greatest centrality. Also from the matrix, the Jaccard distance is calculated between the miRNAs based on their targets, i.e., similarity, and a dendrogram and an identity plot are generated to identify how closely related the miRNAs in the study are. More details about the package installation and dependencies can be found in the package vignette.

To illustrate the usefulness of our method to interpret miRNA–target interactions in a biological application, we used transcriptomic (i.e., mRNA and miRNA) data from the human bladder cancer cell lines T24 (poorly metastatic) and FL4 (its metastatic derivative) [47]. Both datasets are available at the ArrayExpress repository and can be found under the accession numbers E-MTAB-2610 and E-MTAB-2611, for mRNA and miRNA respectively. The processed data and probe-to-gene annotation were downloaded from the ArrayExpress repository, probe IDs were annotated to gene symbols as designated by the Human Genome Organization (HUGO) Gene Nomenclature Committee, and where multiple probes were present for a given gene the highest expression value was selected; finally, differential expression (DE) analysis was performed using Limma [51] Bioconductor R Package version 3.32.10, and a *p* value of 0.05 and a linear fold change of two were used as the threshold for statistical significance. The correlation of miRNA–gene targets for the upregulated DE miRNAs and downregulated DE genes were acquired using the multiMiR [47] Bioconductor R Package, considering only the top 35% of predicted interactions.

## 3. Results

In this section, we demonstrate the usage of miRmapper when applied to biological data and discuss the functionalities of the software and its outputs. We analyzed differential transcriptomic data—miRNA and gene expression—from cell lines T24 and FL4 and built the table with the miRNA–target interaction and DE genes (Table 2 and Table 3 and Supplementary Tables S1 and S2).

Two data frames containing these data are available within the package. The template data are loaded into an R environment as follows:

R > data (“interaction.matrix.miR.up”)

R > interact <- (interaction.matrix.miR.up)

R > data (“DE.gene.dn”)

R > DEgene <- (“DE.gene.dn”)

The input tables, as they contain all the information of the miRNA–gene target network, have a size that do not allow the researcher to interpret it. It is necessary first to organize it in a way that enable it to be read. We first generate a mirMapper object, as described below:

R > miRm <- miRmapper (interactions = interact, DEgenes = DEgene)

The next step is to generate an adjacent matrix (Table 4 and Supplementary Table S3) using Supplementary Table S1 as input, as described below:

R > adjMat(miRm)

The adjacency matrix provides two results: first, the data organization allows the user to perform downstream analysis; second, it defines the gene targets with the greatest degree of centrality; in this case, the gene Transcription Factor 4 (*TCF4*). *TCF4* (log 2-fold change = −1.22, *p* = 0.001, Supplementary Table S2) has a greater degree of centrality than the serglycin gene, *SRGN* (log 2-fold change = −6.0, *p* = 4.68 × 10^−5^, Supplementary Table S2), the most downregulated transcript that has a degree of centrality = 2 (Supplementary Table S3).

**Figure 3:**
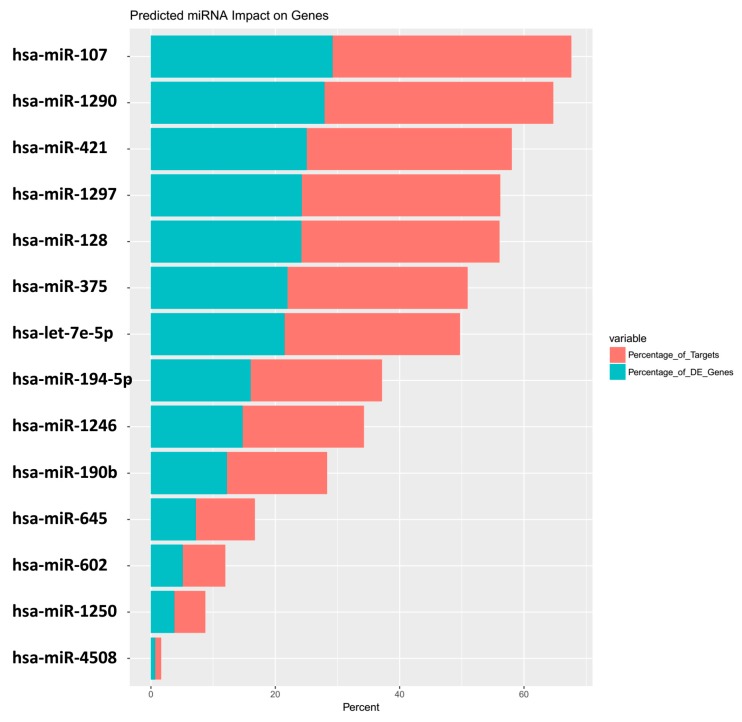
miRmapper output: miRNA boxplot. Data are presented in the order of the greatest number of impacted genes to the lowest, with the percentage of total targets affected by the miRNA in red and the percentage of total DE genes affected by the miRNA in blue.

This matrix is used as input to also define the centrality of the miRNA itself, depicting it as a table (Table 5 and Supplementary Table S4) and graphically in a bar plot (Figure 3a).

The miRNA hsa-miR-146a (log 2-fold change = 4.7, DE p = 6.39 × 10^−5^, Supplementary Table S5), with the greatest fold change, had no impact on its targets, whereas hsa-miR-107 (log 2-fold change = 1.4, *p* = 0.007), with a linear expression 8 times smaller than miR-146a, has an impact on 29.21% of all downregulated genes and regulates 38.41% of all the genes being targeted by a miRNA in the dataset (Table 5).

The miRNA impact matrix (Table 5 and Supplementary Table S4) can be made using the command:

R > getImpact(miRm);

and the barplot (Figure 3) with the command:

R > barPlot(miRm).

**Figure 4:**
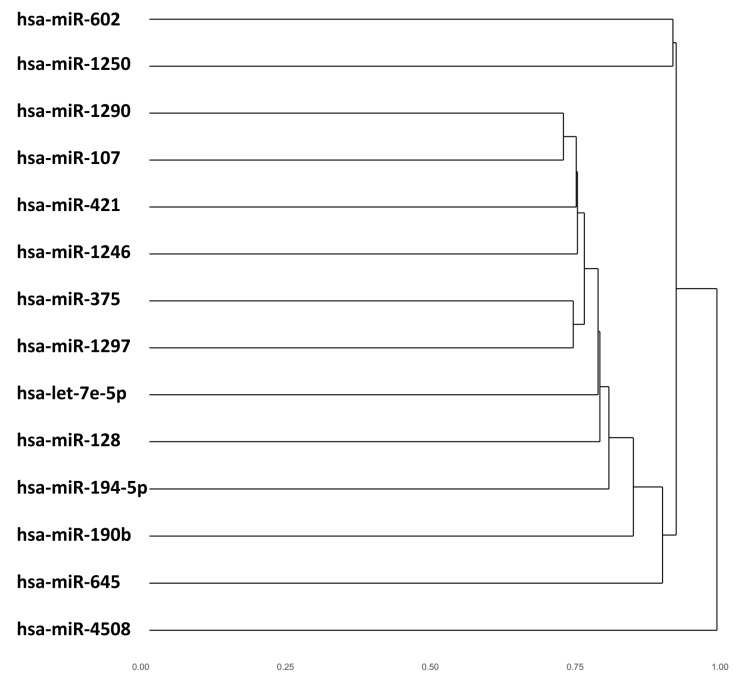
miRmapper output: dendrogram. The cluster is based on the similarity of the miRNAs’ Jaccard index values to each other.

**Figure 5:**
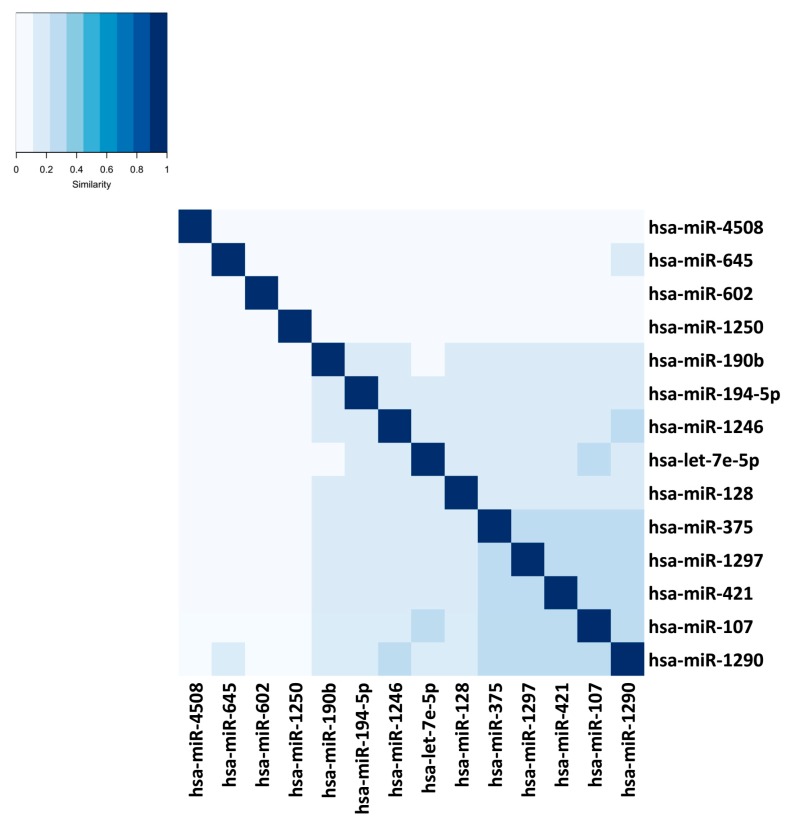
miRmapper output: identity plot with the miRNAs clustered by mRNA target similarity. The distances were based on the similarity of the miRNAs’ Jaccard index values to each other.

The miRmapper approach allows the user to rapidly identify those miRNAs which are working synergistically (Figure 4 and Figure 5), as it is normally necessary for more than one miRNA to act on a target to cause a significant impact in the transcript levels [52]. In our case, we found that hsa-miR-107, hsa-miR-1290, hsa-miR-421, hsa-miR-1297, and hsa-miR-375 were clustered as having similarly modulated mRNA targets, which allows us to infer that they are working cooperatively. These five miRNAs belong to five distinct miRNA families [53], and we would not be able to infer that they are working together with their sequence analysis only.

The dendrogram (Figure 4) can be made using the command:

R > dendrogram(miRm);

and the identity plot (Figure 5) with the command:

R > identityPlot(miRm).

The package has the capability of running all of the above functions at the same time and saves the outputs in the working directory using the function below:

R > runAnalysis(miRm).

## 4. Discussion

When changes occur in a transcriptional network, it is not only important to know which genes are changed the most with regard to the level of their gene expression, but also which are the most relevant changes in the context of the network [39]. A miRNA that exhibits greatly upregulated expression between two biological conditions, but with none of its transcribed targets being downregulated, can be seen as a potential biomarker on its own, but this isolationist approach does not demonstrate far-reaching biological relevance. On the other hand, a miRNA that is mildly upregulated, yet causes a deep impact in the downregulation of its targets, would be of global relevance for the cell. In our miRNA DE analysis and miRmapper analyses, we found 31 upregulated miRNAs (Supplementary Table S5), but only 14 of them showed an impact in the downregulation of their targets (Supplementary Table S4).

Our analysis recognized miR-107 as the most important DE miRNA, based on the number of affected targets. miR-107 was shown to promote migration and invasion in osteosarcoma, hepatocellular carcinoma, and pancreatic ductal adenocarcinoma [54,55,56]. There is no report in the literature about the role of miR-107 in bladder cancer; the original analysis of the dataset elected mir-146a as a possible metastasis inducer, and although data of mir-146a in bladder cancer is also scarce, mir-146a overexpression has been reported to inhibit migration, invasion, and metastasis in bladder cancer [57]. The lack of downregulated mir-146a targets in our analysis agrees with the report and provides more support for the hypothesis that miR-146 can have different roles in different tissue types [58].

Similarly, our analysis recognized *TCF4* as the most regulated gene. Although TCF4 was reported to promote cancer cell stemness and metastasis in breast cancer patients [59,60] and in clear cell renal cell carcinoma [61], its role in invasive bladder cancer was described to be beneficial, participating in the inhibition of tumor growth [62]. This can be an indication that, as with miR-146a, the roles of TCF4 are tissue-specific.

We also identified hsa-miR-107, hsa-miR-375, hsa-miR-421, hsa-miR-1290, and hsa-miR-1297 as working synergistically. Although hsa-miR-375, hsa-miR-1290, and hsa-miR-1297 were identified to target the same transcription factor cluster [63], the literature has no report of these five miRNAs influencing gene expression together and of their roles in bladder cancer. miR-375 was shown to play a role in epithelial-to-mesenchymal transition and in the recurrence of breast cancer [64,65]. miR-421 was found to induce cell migration and metastasis in neuroblastoma, osteosarcoma, and gastric cancer [66,67,68]. In the context of breast cancer, both were described as capable of inducing and inhibiting metastasis [69,70], emphasizing again the context-dependent role of regulatory elements in gene expression. hsa-miR-1290 was demonstrated to have a role in cancer stem cell formation and metastasis in non-small cell lung cancer [71], to promote metastasis in esophageal squamous cell carcinoma [72], and as a prognostic marker for a poor outcome in colorectal cancer [73]. As with miR-421, miR-1297 was reported to induce migration and invasion of colorectal cancer cells [74], but to inhibit invasion in prostate and hepatocellular carcinoma [75,76].

Both miR-107 and *TCF4* are part of the Wnt signaling pathway [62,77]. The Wnt pathway plays a key role in regulating development and stemness and pathway members are typically altered in aggressive cancers, including bladder cancer [78,79]. Considering also the description of the synergistic properties of hsa-miR-107, hsa-miR-1290, hsa-miR-421, hsa-miR-1297, and hsa-miR-375, we, for the first time, identified a possible pivotal axis in the development of bladder cancer metastasis that can be tested in the laboratory. This discovery was only made possible through the use of our tool, miRmapper, which allowed evaluation of network topographic properties of miRNA–mRNA target networks in a simple and visual way.

## 5. Conclusions

The miRmapper tool identifies the most dominant miRNAs in a miRNA–mRNA network and recognizes functional similarities between miRNAs based on their commonly regulated mRNAs.

The miRmapper software uncovers novel cooperative interactions between miRNAs from independent families in regulating common target mRNAs. We showed here that miRmapper identified miRNAs and regulated mRNAs involved in a known pathway for cancer metastasis, i.e., the Wnt signaling pathway. This highlights the utility of miRmapper to interpret miRNA–gene networks and to identify key elements and possible biomarkers and drug targets. Future improvements of the methodology will address noncanonical miRNA functions. The source code of the package and the tutorials are available on GitHub at http://github.com/ MUSC-CGM/miRmapper. Installation documentation and a detailed vignette are provided.

## Figures and Tables

**Table 1:** Tool comparison. Each column represents a feature and each row represents a software tool.

Tools	Input Your Own Data	Output Contextualized with Your Experimental Design	Calculate the Centrality of miRNAs in the Network	Calculate Centrality of Genes in the Network	Calculate the Structural Equivalence of miRNA Interactions	Graphical Depiction of miRNAs Organized by Centrality	Graphical Depiction of miRNA Clusters by Structural Equivalence
miRmapper	X	X	X	X	X	X	X
MMIA	-	-	-	-	-	-	-
miRror-Suite	X	X	-	-	-	-	-
DIANA-mirExTra	X	X	-	-	-	-	-
miRGator	-	-	-	-	-	-	-
MAGIA	X	X	-	-	-	X	-
MAGIA^2^	X	X	-	-	-	X	-
NetworkAnalyzer	X	X	X	X	-	-	-
SpidermiR	-	-	X	X	-	X	-

MMIA: MicroRNA and mRNA integrated analysis

**Table 2:** miRmapper input. miRNA-gene interaction data frame, no headers.

hsa-miR-107	*N4BP1*
hsa-let-7e-5p	*FNDC3A*
hsa-let-7e-5p	*HAND1*
hsa-let-7e-5p	*IGF1R*
hsa-let-7e-5p	*OSBPL3*
hsa-let-7e-5p	*RRM2*
hsa-let-7e-5p	*STX3*
hsa-miR-107	*ASH1L*
hsa-miR-107	*CAPZA2*
hsa-miR-107	*YWHAH*
hsa-miR-421	*AFF4*
…	…

**Table 3:** miRmapper inputs. List of total differentially expressed genes; this is an optional input.

*IFI16*
*COL5A2*
*GJA1*
*ALCAM*
*TXNIP*
*PLS3*
*CXCL8*
*SPARC*
*FBN1*
*CDH2*
*TMEM158*
…

**Table 4:** Adjacency matrix of top 10 regulated genes.

	hsa-miR-107	hsa-let-7e-5p	hsa-miR-421	hsa-miR-1297	…	Sums
*TCF4*	1	1	1	1	…	12
*FNDC3A*	1	1	1	1	…	10
*ZFHX4*	1	1	0	1	…	10
*IGF1R*	1	1	1	0	…	9
*RPS6KA3*	1	1	1	0	…	9
*PDE4D*	1	0	1	1	…	9
*ELL2*	1	1	1	1	…	9
*MAPK1*	1	1	0	1	…	9
*EXOC5*	1	1	0	1	…	9
*CCDC6*	1	0	1	1	…	8

The interaction between a miRNA and gene is depicted as binary: “1” means the gene is a target for the miRNA; “0” means it is not.

**Table 5:** miRNA impact on the gene expression; upregulated miRNA affecting downregulated genes.

miRNA	Predicted_Genes_Found	Percentage_of_Targets	Percentage_of_DE_Genes
**hsa-miR-107**	373	38.4	29.2
**hsa-miR-1290**	357	36.8	28
**hsa-miR-421**	320	33	25.1
**hsa-miR-1297**	310	31.9	24.3
**hsa-miR-128**	309	31.8	24.2
**hsa-miR-375**	281	28.9	22
**hsa-let-7e-5p**	274	28.2	21.5
**hsa-miR-194-5p**	205	21.1	16.1
**hsa-miR-1246**	189	19.5	14.8
**hsa-miR-190b**	156	16.1	12.2

DE: Differentially expressed

## Supplementary Materials

The following are available online at http://www.mdpi.com/2073-4425/9/9/458/s1: Supplementary Table S1: input list of miRNA–DE gene target, Supplementary Table S2: input complete list of DE genes, Supplementary Table S3: complete output of adjacency matrix, Supplementary Table S4: complete miRNA impact on the gene expression, Supplementary Table S5: complete upregulated DE miRNA.
